# Ensemble learning approach for advanced metering infrastructure in future smart grids

**DOI:** 10.1371/journal.pone.0289672

**Published:** 2023-10-18

**Authors:** Muhammad Irfan, Nasir Ayub, Faisal Althobiani, Sabeen Masood, Qazi Arbab Ahmed, Muhammad Hamza Saeed, Saifur Rahman, Hesham Abdushkour, Mohammad E. Gommosani, V. R. Shamji, Salim Nasar Faraj Mursal

**Affiliations:** 1 Electrical Engineering Department, College of Engineering, Najran University, Najran, Saudi Arabia; 2 Department of Creative Technologies, Air University Islamabad, Islamabad, Pakistan; 3 Faculty of Maritime Studies, King Abdulaziz University, Jeddah, Saudi Arabia; 4 Department of Software Engineering, Capital University of Science and Technology Islamabad, Islamabad, Pakistan; 5 Department of Software Engineering, University of Azad Jammu and Kashmir, Muzaffarabad, Pakistan; 6 Department of Computer Science, National Textile University, Faisalabad, Pakistan; 7 Nautical Science Department, Faculty of Maritime Studies, King Abdulaziz University, Jeddah, Saudi Arabia; University of Sargodha, PAKISTAN

## Abstract

Typically, load forecasting models are trained in an offline setting and then used to generate predictions in an online setting. However, this approach, known as batch learning, is limited in its ability to integrate new load information that becomes available in real-time. On the other hand, online learning methods enable load forecasting models to adapt efficiently to new incoming data. Electricity Load and Price Forecasting (ELPF) is critical to maintaining energy grid stability in smart grids. Existing forecasting methods cannot handle the available large amount of data, which are limited by different issues like non-linearity, un-adjusted high variance and high dimensions. A compact and improved algorithm is needed to synchronize with the diverse procedure in ELPF. Our model ELPF framework comprises high/low consumer data separation, handling missing and unstandardized data and preprocessing method, which includes selecting relevant features and removing redundant features. Finally, it implements the ELPF using an improved method Residual Network (ResNet-152) and the machine-improved Support Vector Machine (SVM) based forecasting engine to forecast the ELP accurately. We proposed two main distinct mechanisms, regularization, base learner selection and hyperparameter tuning, to improve the performance of the existing version of ResNet-152 and SVM. Furthermore, it reduces the time complexity and the overfitting model issue to handle more complex consumer data. Furthermore, numerous structures of ResNet-152 and SVM are also explored to improve the regularization function, base learners and compatible selection of the parameter values with respect to fitting capabilities for the final forecasting. Simulated results from the real-world load and price data confirm that the proposed method outperforms 8% of the existing schemes in performance measures and can also be used in industry-based applications.

## Introduction

Market operations in electrical energy networks include determining projected electricity prices and the load demand over a long period. Market participants rely on electric price prediction values to devise strategies for broadcasting buy-and-sell bids for power selling and buying in trading energy in the community. Electricity price bidding requires the computation of a price in real time. For entities to engage in the bidding process, accurate forecasting of power costs is necessary. Knowing electricity prices over a longer horizon is crucial for the coming day’s market to select the units to be somehow devoted, referred to as cost-based unit allegiance, to offer available power production across the operating situation. Over the years, significant utilities have dominated the electric power business, with all-inclusive control operations in power production, transferal and energy distribution within their operation zone. Vertically integrated utilities is a term used to describe such utilities. These utilities were the sole power source in the area and were required to supply electricity to anybody [1]. Due to the vertical integration of the utilities, it has frequently been impossible to separate the costs of production, transmission and distribution. As a result, utilities usually charge their users an aggregate price based on their overall cost over time. An external regulatory agency determined the prices, which typically considered factors other than economics. Due to the apparent ongoing liberalization of the power business, wholesale power markets have rapidly expanded worldwide. The electricity generation and transmission industries are separated. As a result, power trading has become a critical component of the power business [2].

Peak power monitoring is used in the construction of new energy systems. Precision forecasting of prospective load across multiple periods is required for the responsible and effective management, planning and dispatch of energy systems. The Smart Grid (SG) was designed to solve the above-mentioned challenges. The conversion to SG can be done by incorporating the two-way communication strategy in the traditional grid. The SG network architecture can efficiently manage energy transmission and energy production. Transmission is a standard electrical mechanism that allows consumers and utilities on both ends to interact. Energy is a resource that is both needed and precious. Consumers in this sector are compelled to shift their load usage in response to changing price rates over time [3].

**Fig 1:**
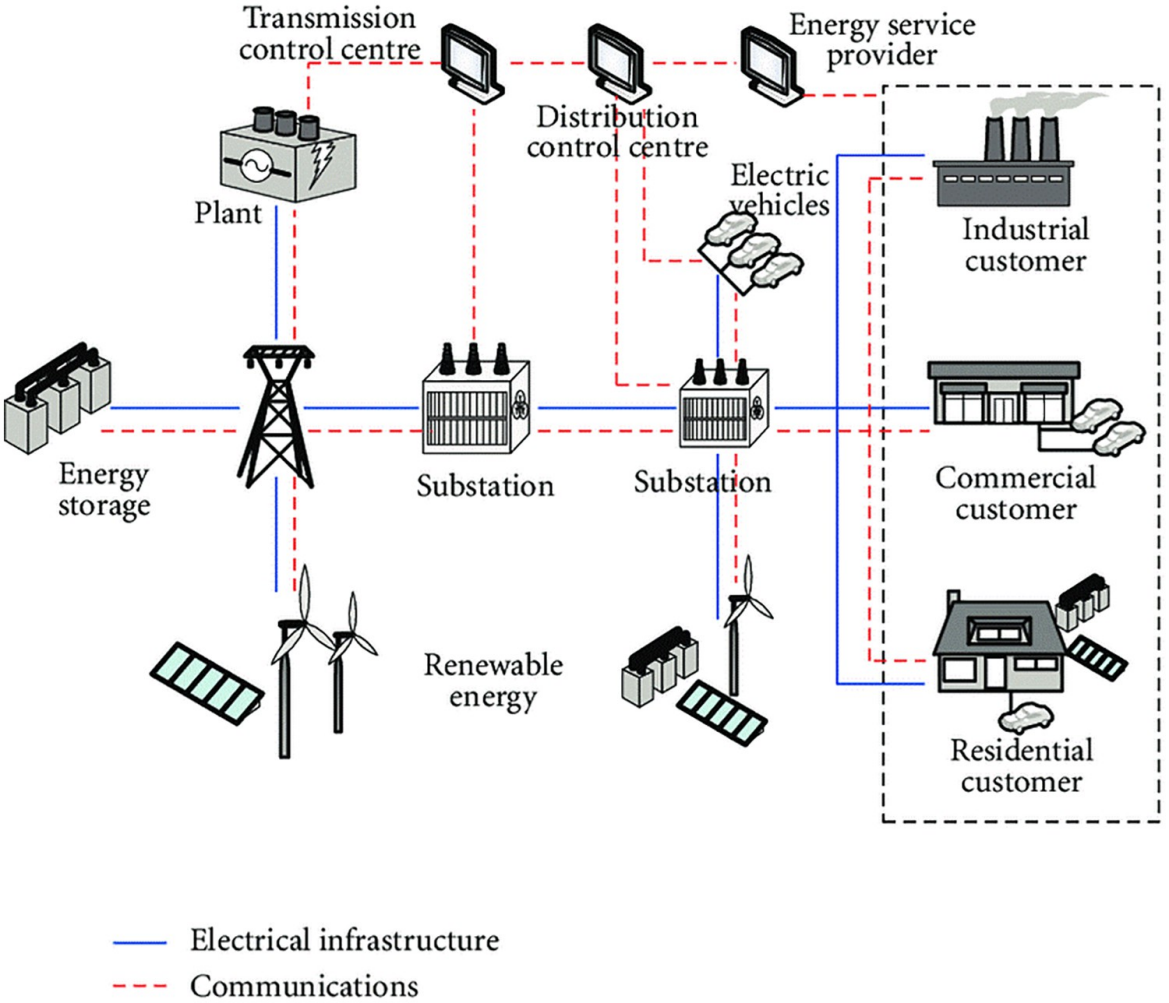
SG domain (abstract diagram).

Demand Side Management (DSM) is a crucial SG function that helps to reduce power utilization costs. It enables energy providers to lower peak load consumption. The proposed solution is based on current system identification and allows users to plan their loads. SG helps consumers achieve efficiency and sustainability by providing large amounts of energy storage. Suppose the suppliers and customers are encouraged to share their real-time information. In that case, the Smart Meter (SM) makes the strategy feasible for collecting pattern information regarding the upcoming energy generation. This aids in maintaining the energy consumption and production balance by moving demand into the low peak hours from the high peak hours and preserving resources; the customer uses SG services and saves time and money. Fig 1 depicts an abstract representation of the SG domain [4].

DSM allows customers to track their energy user trends, which depend on the utility’s price. Market competitors profit more from Load Forecasting (LF). Growth, distribution processes, resource, production management, performance monitoring and quality control are all problems to address in light of impending load estimates. Efficient energy generation and utilization is another issue in the electrical business. The customer’s and the utility’s primary objective is to maximize utility. As a result of more precise load estimates, energy producers will incur more significant expenses, while consumers will benefit from lower power rates. Accurate LF has become increasingly important from the viewpoint of market rivals [5]. The Independent System Operator of the New England Control Area (ISO-NE) is a distinct power grid-managed local distribution system. In place to handle the activities of the domestic electricity sector. It serves the states of Vermont, Connecticut, Rhode Island and Massachusetts in New England. The work in this article utilizes a large dataset of consumers of different cities in NE Temperature, weather conditions, and other factors all impact electrical load.

Thus pricing isn’t the sole issue. Many relevant, reliable data and information from the SG are examined. A good decision process in the end-user minimizes power loss, decreases energy costs and reduces Peak Average Ratio (PAR). Because of these problems, researchers are concentrating their efforts on the issue of power scheduling.

Understanding the behavior of consumers’ consumption, according to the variation in the pricing rates, is difficult. Energy market decisions depend on the energy demand of the consumer. Using novel models for predicting the energy demand with the variation in pricing rates is still an unresolved issue [6]. Price forecasting (PF), unlike LF, has received less attention. Participating customers must be able to successfully change their load tariff in response to forecasted data [7]. The author in [8] offered a complete evaluation of PF after learning about its importance. In a deregulated environment, a nonlinear model was designed to solve the challenge of calculating the best power price. In a pool-based energy market, the author explored the elasticity of demand with generation scheduling and price-fixing. The author in [9] created a mixed load and pricing forecasting model based on an iterative Neural Network (NN) model. The author used a multi-input multi-output engine with a based association mining method that considered bidirectional relationships to develop initial demand and price projections.

To resolve these issues, a classification with a learning-based approach is created. Furthermore, model parameters are optimally tuned with the metaheuristic algorithm to achieve maximum accuracy. Extreme Gradient Boosting (XGBoost) and Random Forest (RF) approaches are used in the Feature Engineering process to remove duplication and optimize the data. Residual Network (ResNet-152) and Support Vector Machine (SVM) are applied for the forecasting along with the optimization method Hunger Games Search (HGS), which is used to provide the optimal values for the model hyperparameter. The updated SVM and ResNet152 networks have the benefit of being able to handle a large amount of input data. This paper’s key contribution is:

- We reduced the computational time and intricacy of the proposed system model training by choosing the most significant features. An ensemble feature selection method is presented to ensure adaptability, integrating RF and XGBoost and feature extraction with Recursive Feature Eliminator (RFE). These methods are sorely tested upon different datasets.
- An improved classifier, ResNet-152 and SVM are tuned with HGS by defining a subset of its parameters to enhance model training accuracy, minimize model loss, reduce non-linearity and overfitting problems and use the required resources to reduce computation time.
- Our proposed classifiers are compared against existing schemes using performance metrics, including Mean Average Percentage Error (MAPE), Accuracy, Mean Square Error (MSE), recall, prediction, Root Mean Square Error (RMSE), f-score, Mean Average Error (MAE), and statistical analysis tools (Pearson test, ANOVA test, students test, chi-square test, etc.). The suggested techniques (SVM-HGS and ResNet-152-HGS) outperformed the existing mechanisms (LR [10], LDA [11], SVM [12], and GWDO [13]) in attaining prediction accuracy, low error rate and low computational time.

## Literature review

The advent of an SG implies more comprehensive load modeling that considers individual appliance behavior [14]. A study of household power usage in the United States discovered that 66% of the energy (out of the total) is consumed by space heating, water and air conditioning [15]. Buildings save significant amounts of power if correctly constructed, designed and operated. As a result, the efficiency of the building energy may be a critical component in addressing carbon emissions, energy shortages and the high threat they impose on our living environment. This study reviews buildings’ energy usage, appropriate energy-saving strategies, and global climate change impact. Data from international energy reports are used to compare energy consumption circumstances in the United States, China and the European Union. The current top three building energy consumers have parallels and distinctions regarding the energy fuel types and end-users.

Top-down, bottom-up, or hybrid load profile modeling can be used in residential controls [16]. The top-down paradigm does not consider individual household usage, which treats the residential load as a huge energy pool. In a bottom-up method, data from each appliance is considered, whereas the hybrid model combines top-down and bottom-up characteristics [17]. The top-down model is explored in [18] and is found to be acceptable for determining supply-side requirements since it employs previous measures and does not account for future changes in load. The data from residential, commercial and industrial consumer meters are combined for load modeling [19]. Data noise and model misspecification are distinctly identified. However, the author has not handled the complexity and non-flexibility of the model.

Customers’ responses and grid dependability are critical in realizing an SG’s benefits, which leads to supply suppliers and end-users making informed decisions. The author forecasts the load with machine learning methods and features engineering methods to retain the supply balance. Furthermore, the complexity is condensed by selecting relevant features, [20] allowing users to make electricity load plans and save money. The electrical load may be split into two sections in a hybrid model: a scaled curve load with five Artificial Neural Networks (ANNs) and day maxima and minima with fuzzy logic and ANNs for LF. The suggested hybrid structure had the advantage of using fuzzy logic and ANNs to handle uncertainty [21]. The noise in data and misspecification are distinctly identified. The model can forecast correctly; however, the drawback is that the model is too complex and non-flexible. A hybrid solution for daily LF is proposed in [22], which uses an NFN with an enhanced Genetic Algorithm (GA). Fuzzy logic was employed to cope with fluctuating linguistic information in LF, and the GA was used to determine the best policy of fuzzy rules. The frequent convergence issues in maxima and minimum approaches have been limited to this method’s initial values. For short-term LF (STLF), an expert system called LoFy was created. It comprises three models for forecasting: day, weekend and special days.

For STLF, another AI approach called Multilayer Perceptron (MLP) was used. It contains a data mining mechanism for developing STLF rules. The optimum tree regression method may also establish a link between dependent and independent variables. It’s also beneficial for pre-filtering raw data by grouping it into clusters. As a result of the similarity of categorized input data, MLP is simple to employ hourly load policy planning using time series models. MLP can forecast with low-performance error; however, the model is complex due to the presence of redundant data, which leads to an increase in model loss and a reduction in training accuracy [23]. ANN models can be used with time-series models for improved policy planning of hourly loads for all days. This strategy consists of two elements. One method relies on correlation approaches for selecting input variables and training sets. The identical problem was solved using the DenseNet, and statistical learning theory technique [24]. It predicts many possible power price realizations without accuracy loss for 24 hours. The model training time is maximum due to the presence of redundant data, which is not handed out using feature engineering methods. The DesneNet differs from NNs in that it constructs the linear decision boundaries into the new dimension space with more layers and objective functions as opposed to NNs’ complex nonlinear mapping.

SVMs are used for short-term scheduling [25]. Compared to autoregressive approaches, SVM performs better in predicting since it produces results by evaluating before and current data points while disregarding significant supplementary features. SVM may also be used to calculate the monthly load demand. The author presents a Multilayer NN (MLNN) model with a long processing time for demand forecasting. To improve forecasting accuracy, [26] combines three schemes: cuckoo search, singular spectrum analysis and SVM. The authors calculated the accuracy in [27] using data preprocessing techniques such as feature selection and extraction and removing unnecessary data to anticipate accurate forecasting. Furthermore, the author has used only the Corona herd optimization algorithm to find the electricity load and forecast prices. However, other parameters like computational complexity and sensitivity analysis are not handled.

As a hybrid model of Ant Colony Optimization (ACO), GA is a regularly used technique for feature selection and GA for LF is provided in [28] and Adaptive Neuro-Fuzzy Inference System (ANFIS) is utilized for the best prediction. The model is adaptable and capable of dealing with complicated load signals; however, the performance error is too high. The information regarding the monthly significant-high price levels and the fluctuation in price patterns are also evaluated, adding to this work. In [29], several ANN models are compared regarding forecasting problems concerning the no. of neurons on each layer and some other variables. The clustering of groups is the critical strategy they employ. Clustering, according to their findings, produces 20% better outcomes. The authors use a fuzzy NN, support vector machines and an adaptive wavelet NN. After analyzing the literature review, some issues are addressed from the literature and resolved in our proposed system. The comparison is discussed in Table 1.

**Table 1:** Different parameter comparisons of the existing and proposed system.

Parameter	Existing Literature [7, 18, 21, 22, 26, 29–31]	Proposed System
Dataset	1 to 3 Years (2013-2019)—Old data	4 Years (Jan 2018–Sept 2022) Updated features
Consumer type	All consumers are treated the same	Consumers are split into high and low consumption
Optimization Algorithm	Only used by [30] Coronavirus herd optimization algorithm	Hunger Games Search Optimizer (Designed in 2020)
Classifier	Simple NNs	ResNet with 152 Layer
Accuracy of Model	84% to 90% on price and 88 to 93% on load	93.159% on cost and 98.35% on load data
Efficient	90%—94%	97%
Computational Complexity	Not computed	Very less among all techniques
Sensitivity analysis	Not computed	Computed
Focus on Data Spikes	Not considered	Considered for both high and low consumers
Data Handling	If data increases, computational time along with overfitting occur for simple CNN	ResNet, with 152 layers, can handle big data with low computational cost
Data Prediction capacity	Yearly data is changed and the proposed algorithm may not be able to classify the upcoming data as the proposed algorithm is not trained on the newly updated pattern data.	The proposed model can be used for the current upcoming year data as we have applied the updated dataset till 31st Dec 2021.

The novelty of this article lies in its innovative approach to address multiple challenges in LF and PF forecasting as discussed below:

- Computational Complexity: The paper tackles the challenge of computational complexity in load forecasting. By proposing enhanced versions of ResNet-152 and SVM models, the study achieves remarkable accuracy while minimizing the computational time required. This contribution is crucial as it enables efficient and scalable load forecasting, even when dealing with substantial amounts of data.
- Non-Linearity: Addressing the issue of non-linearity in load forecasting, the paper utilizes ResNet-152 and SVM models renowned for their ability to handle complex relationships. Consequently, the proposed approach accurately captures and models intricate patterns within the load data. This contribution significantly improves forecasting accuracy, particularly in scenarios where non-linear relationships play a pivotal role.
- Optimal Parameter Values: Introducing the HGS optimizer, the paper facilitates optimal parameter value selection for the classifier models. Leveraging this optimizer, the proposed models exhibit improved performance in terms of training accuracy, reduction in model loss, and overall forecasting accuracy. This contribution enhances the effectiveness and efficiency of the load forecasting process.
- Overfitting Problem: The paper addresses the challenge of overfitting, which often leads to poor generalization and inaccurate forecasts. By incorporating mechanisms like regularization and meticulous base learner selection, the proposed models effectively mitigate overfitting concerns, resulting in improved generalization capabilities. This contribution enhances the reliability and robustness of the load forecasting models.
- Feature Engineering and Selection: Emphasizing the significance of feature engineering and selection, the paper employs techniques such as XGBoost, RF, and RFE to handle missing values, redundant features, and irrelevant attributes within the input data. Consequently, the proposed approach enhances the quality of the input data, leading to improved overall forecasting accuracy.
- Stabilization Study: The paper conducts a comprehensive stabilization study that assesses the performance of the recommended models across varied data sizes. This study ensures the stability and reliability of the proposed models across different data scenarios. The inclusion of this study adds scientific rigor to the research, strengthening confidence in the proposed approach.
- Integration of Online Learning: To overcome the limitations of batch learning in load forecasting, the paper introduces an innovative online learning method. This approach enables the load forecasting model to adapt effectively to real-time data, ensuring better integration with the dynamic procedures of ELPF.
- Improved Models: Building upon existing techniques, the paper presents enhanced versions of Residual Network (ResNet-152) and Support Vector Machine (SVM) models for load forecasting. These models incorporate regularization, base learner selection, and hyperparameter tuning to boost their performance. Through comprehensive simulations and comparisons, the proposed ResNet-152 and SVM models exhibit superior precision and outperform 8% of existing approaches in various performance measures.
- Handling Complex Consumer Data: The proposed models effectively address the challenges associated with non-linearity, unadjusted high variance, and high dimensions in load forecasting. By mitigating time complexity and addressing overfitting issues, these models demonstrate the capability to handle complex consumer data. This adaptability makes them well-suited for practical applications in industry settings.
- Real-world Validation: To validate the proposed method, the paper employs real-world load and price data. The simulated results confirm the superiority of the proposed approach, validating its effectiveness in practical Electricity Load and Price Forecasting (ELPF) scenarios.

## Proposed system model

This paper introduces two working models, electricity LF and electricity PF. Both models use the same strategies, as these models are related. One model is used to anticipate power demand, while the other is used to determine the price of electricity. The fundamental purpose of this study is to optimize the energy load and the accuracy of price predictions, as all the users do not have the same consumption pattern. The data in the dataset is divided into high and low users based on consumption to identify the exact pattern of consumers. We have considered both high and low users in our proposed model.

**Fig 2:**
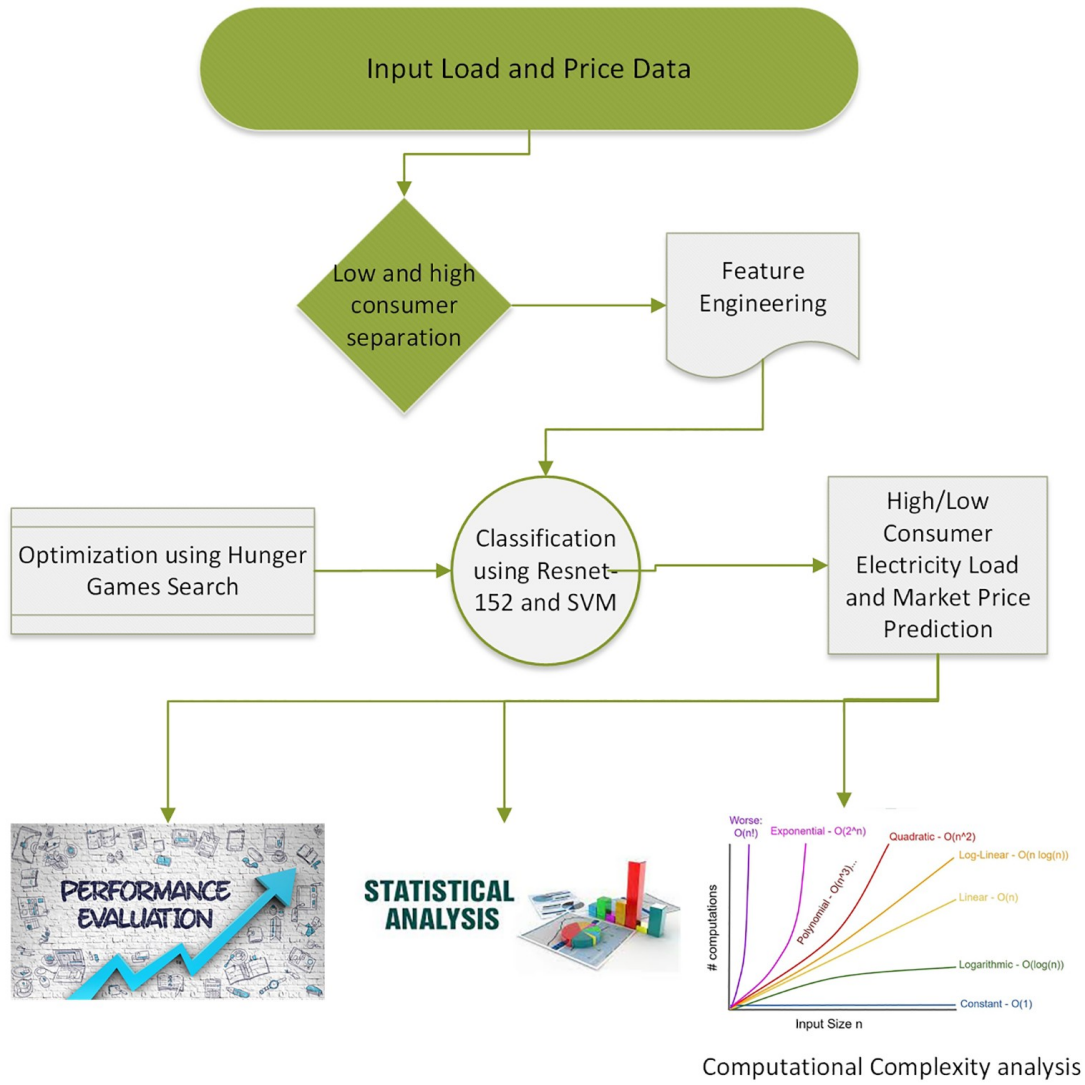
Abstract view of flowchart.

**Fig 3:**
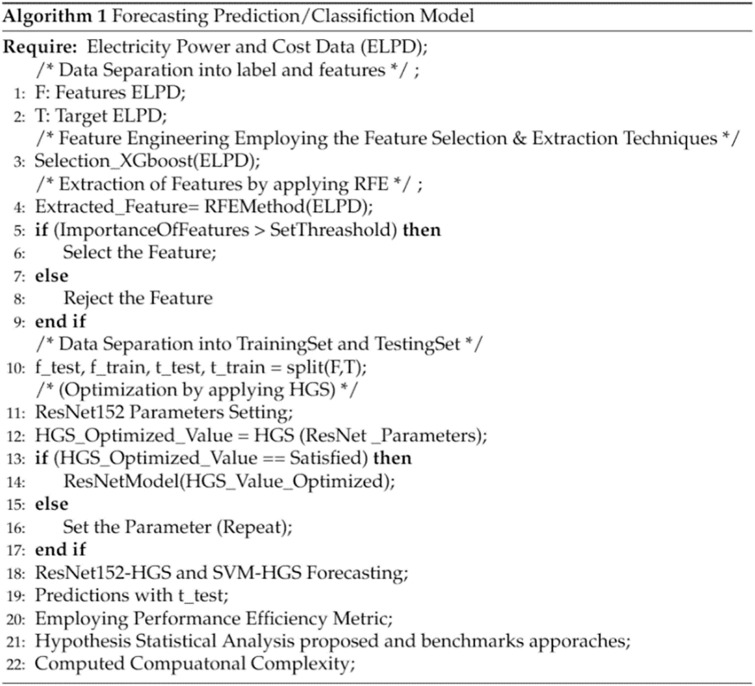
Prediction algorithm.

The proposed model and the forecasting algorithm are described in Figs 2 and 3. After examining the preceding methodologies and literature for predicting the load based on an average selection of features, feature extraction and prediction/forecasting, we propose a novel framework. The feature extraction is done with RFE, while the feature processing is done with XGBoost and RF. The average feature selection values for XGBoost and RF are very close. The mean value of XGBoost and RF is employed to pick features, as shown in Eq 1 for average feature selection.

### Data collection / dataset

This article employs historical data of energy load and price information obtained from various cities in New England. The Independent System Operator of New England (ISONE) is the source of power load information in this study. The data consists of electricity load and price data collected over four years, from January 2018 to December 2021. The data is organized on a monthly basis, and training the model on a monthly basis results in an improved performance and learning rate. The dataset includes 14 different features, with the “System Load” serving as the target feature for prediction. To evaluate the performance of the proposed model, 70% of the dataset was used for training and the remaining 30% for testing purposes.

### Feature engineering

Feature engineering is the process of selecting and extracting features. The RF and XGBoost machine learning approaches are utilized to choose relevant characteristics [14]. The influence of attributes on the target is computed using these methods. The range of 0 and 1 is used for feature importance calculation. As indicated in Eq (1), the average feature importance is used to improve feature selection. This feature extraction process eliminates extraneous features by providing precise and usable characteristics for training on the data. $Fe=\frac{\text{Fimp}(XGB)+\text{Fimp}(RF)}{2}$ is used to take the average of the feature engineering methods. Fimp and Fe stand for feature importance and feature selection, respectively.

Extraction of features is a technique for removing certain dataset features to reduce the complexity. This method uses a subset of the data to offer more specific information than the raw data. (new data is generated from the old data). Various approaches for extracting features from data have been recommended in the different articles. To decrease the training phase complexity during classification, the proposed model uses the RFE, and RF approaches to eliminate the most redundant features. Using positive integers and true/false, the RFE approach sets the priority and dimension of features.

$$\begin{array}{c} \;\text{Fos}\;=\sum_{fr=0}^{n}\{\begin{array}{cc} \;\text{SelectFeature,}\; & {\text{avg}}_{\text{imp}\;}\left(\;\text{feat}\;\right)\geq \alpha \text{RFE}{\left(\;\text{feat}\;\right)}_{pr}\leq \beta pr\\ \;\text{RejectFeature,}\; & {\text{avg}}_{\text{imp}\;}\left(\;\text{feat}\;\right)\geq \alpha \text{RFE}{\left(\;\text{feat}\;\right)}_{pr}>\beta pr \end{array} \end{array}$$ (1)

To remove unnecessary features, the drop-out rate has been computed. Suppose the average feature weight/importance exceeds the defined threshold, and the priority exceeds the stated priority threshold. In that case, features are reserved/selected, according to Eq (1). Features with a lower weight are discarded, while those with a higher weight are chosen. Using the average algorithm, the degree of feature extraction is 0.6. In addition, features with a priority of 5 or higher are considered in the decision-making process. We used Eq (1) to help select the overall feature. It represents the overall feature selection, whereas f represents the particular feature. The terms avg imp and pr are average feature importance and priority, respectively. The selection and extraction methods indicate the levels of feature significance and priority. After feature identification and categorization, the classifier obtains the most relevant features for predictions/forecasting.

### ResNet-152 architecture

ResNet is a type of ANN inspired by the brain cortex’s pyramidal cell architecture. The remainder of the NN employs skip connections, also known as shortcuts, to hop between layers. Standard ResNet models include two- or three-layer jumps with non-linearity (ReLU), and central batch normalization [16]. You may learn the weights of leaps by using extra-weight matrices. These types are known as DenseNets. DenseNets are models with numerous parallel jumps. Non-residual NNs are regarded as simple networks compared to residual N.N.s.

There are two primary reasons for adding/removing connections. To prevent a drop in gradient or the deterioration (accuracy saturation) problem when adding layers to a correctly deep network increases training mistakes. Weights are altered during your workout to mitigate and boost the previously missed layer. Only the connection weights of surrounding levels are changed in the most straightforward instance, with no explicit weighting of the upstream layers. This method works well when a single nonlinear layer is skipped, or the intermediate layers are all linear. Otherwise, if the connection is lost, it must explicitly train the weight matrix (HighwayNet). Skipping layers early in training reduces the number of layers used and simplifies your network. Fewer layers are transmitted to decrease gradient fading effects and speed up learning. The network will recover skipped levels as it learns the functional space. Raise all layers after training to come closer to the manifold and learn more quickly. NNs may explore larger feature areas when the remaining components are removed. This increases the likelihood of bumps and necessitates more training data to exit and recover from the manifold. ResNet’s capacity to train incredibly deep NNs with over 150 layers is a significant addition. The gradient vanishing problem made training an extremely deep NN to ResNet problematic. An input variable x is introduced to the output after several weighting levels, including a skip/join link to address the gradient disappearance/explosion problem.

**Fig 4:**
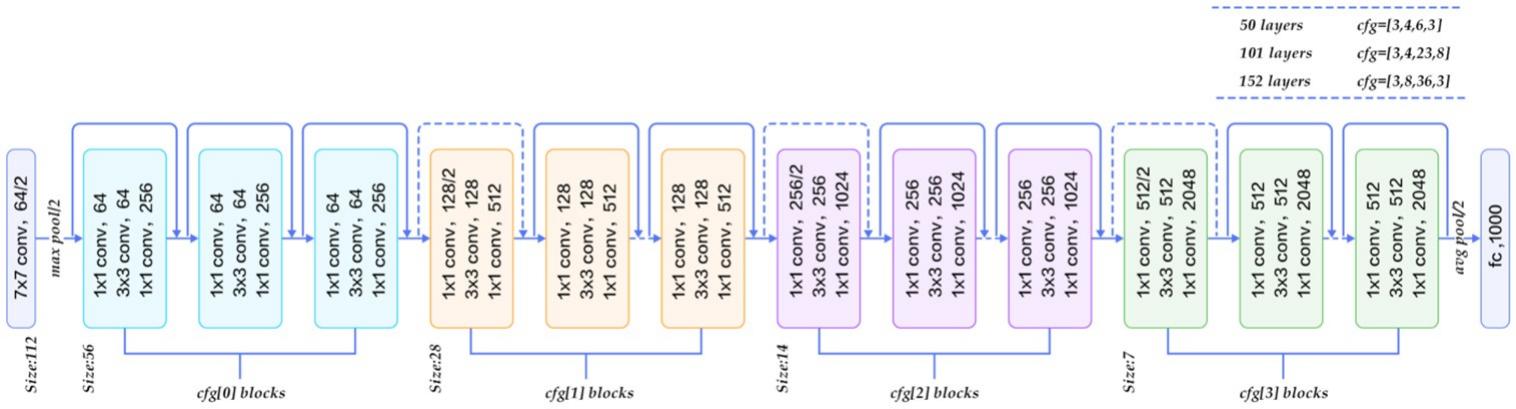
Entire ResNet-152 architecture [19].

As a result, the result is H(i) = F(i) + i. i is the input weight. The weighted layer develops a residual mapping of F(i) = H(i)-i. Even if the weight layers have a decreasing gradient, the identity of variable x is reassigned to prior levels. The VGG-19 [19] (bottom) is a cutting-edge method. The VGG-19’s deeper network, with more conv layers, is the plain network of 34 layers (middle). The 34-layers core network (ResNet) also has a bypass connection (above). In the model of ResNet, two types of bypass links where the initial input sizes are very less than the size of the output. 1) Perform identity mapping with additional null padding to extend the dimension. This means that no further configuration is required. 2) Each shortcut is a projection of the previous shortcut. The additional parameters are greater than the number in (1). The temporal complexity is large since the network is now quite deep. This method is proposed by GoogLeNet and Network (Inceptionv1). 1x1 Conv proves that the number of connections, i.e., parameters, can be minimized while maintaining network performance. The bottleneck design transforms a 34-layered ResNet network into a 50-layered ResNet network. ResNet152 and ResNet101 are two deeper networks with bottleneck configurations. Fig 4 depicts the overall network architecture.

### Support vector classifier tuned with hunger games search

In machine learning, SVM is employed as a supervised learning approach for regression and classification. The primary purpose of SVM is to discover an n-dimensional hyperplane [21]. The number n denotes the number of distinct features. The data is classified using hyperplanes. SVMs are characterized by two properties: SVR for regression and SVC for classification. This study proposes a better SVR by dynamically optimizing parameters with HGS. A collection of parameters inside the range of SVM parameters is referred to as a subset. Table 2 shows all potential parameter combinations that may be represented using this subset following feature selection and extraction. After each variation is added to the classifier, to ensure the accuracy of the classifier’s predictions, the goodness of fit of the combination is determined and various cross-validation procedures are performed.

**Table 2:** SVR parameters for HGS.

Name of Parameter	Values Defined	Parameters Description
kernel	[’poly’, ‘rbf’, ‘linear’]	SVM Kernel
C	[5, 3, 15, 10, 30, 20]	C is a proportion that governs the balance around inadequate training and low testing errors.
gamma	[‘gamma’,‘scale’,20,10,30]	The opposite of the standard deviation is used to equate two lines.
epsilon	[0.2,0.02,0.002,0.0002]	In terms of regressive functional loss, an Error Margin is used to denote.

### Performance evaluation

The models are assessed based on performance metrics: MSE, MAPE, MAE and RMSE. Also, the model is evaluated with sensitivity analysis. Sensitivity analysis examines the influence of each feature on the model’s prediction, which is a simple and powerful approach to analyzing a learning model. To calculate a feature’s sensitivity, change the feature’s value or keep the other features constant and try to ignore it in some way while observing the output of the model. Adjusting the feature value significantly impacts the model’s output, indicating that this feature is essential for prediction [25].

## Experimental setup and simulation

The environment setup for simulation and implementation impacts of our suggested approach in light of error and computational performance indicators are discussed in this section. Our suggested model is implemented on a machine with a Core i7 9th Gen processor, RAM with 8 GB capacity, along with a dual 4.8 GHz processor chip. The IDE of Anaconda (Spyder) and the programming language Python are utilized.

The feature importance obtained by XGBoost and RF ML algorithms is shown in Table 3. The feature significance of a variable indicates how much it impacts the target feature, in this example, the power load. The high significance value of the attribute implies that it has a substantial influence on the intended function. The feature’s high impact indicates its importance to the target. Changes to these crucial characteristics can have a significant influence on the target. Low-importance traits are those that have a low impact. If these qualities are eliminated, the goal has little or no effect. The removal of non-essential features decreases computational costs and simplifies computation.

**Table 3:** Feature importance calculated by feature engineering.

Features	RF Importance	XGBoost Importance	Selection Status
DA_CC	0.700001765	0.7	S
DA_Demand	0.301928891	0.444967574	S
DA_EC	0.300017883	0.433740056	R
DA_LMP	0.700023206	0.779979837	S
DA_MLC	0.70000175	0.783376378	S
Dew_Point	0.300091464	0.435718706	R
Dry_Bulb	0.300031712	0.43533714	R
Reg_Capacity_Price	0.300061195	0.428336932	S
Reg_Service_Price	0.300022106	0.404194175	S
RegCP	0.300054697	0.420705801	S
RT_CC	0.300004072	0.360838203	R
RT_Demand	1.697369103	0.810800982	S
RT_EC	0.300125124	0.430936156	R
RT_LMP	0.700262428	0.781068173	S
RT_MLC	0.300004604	0.35	R

where S Means Selected, and R means Rejected.

**Fig 5:**
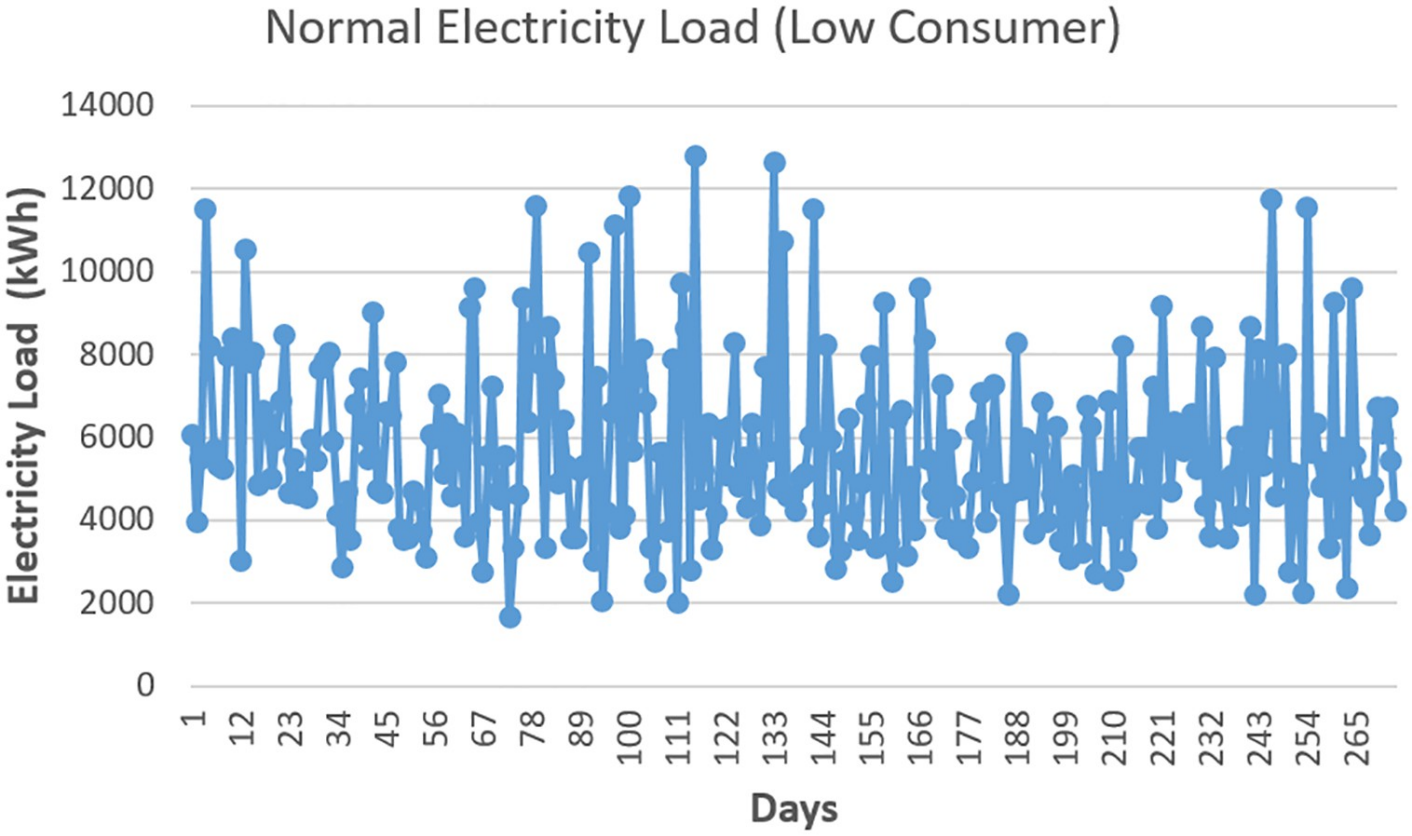
Low consumers electricity load.

**Fig 6:**
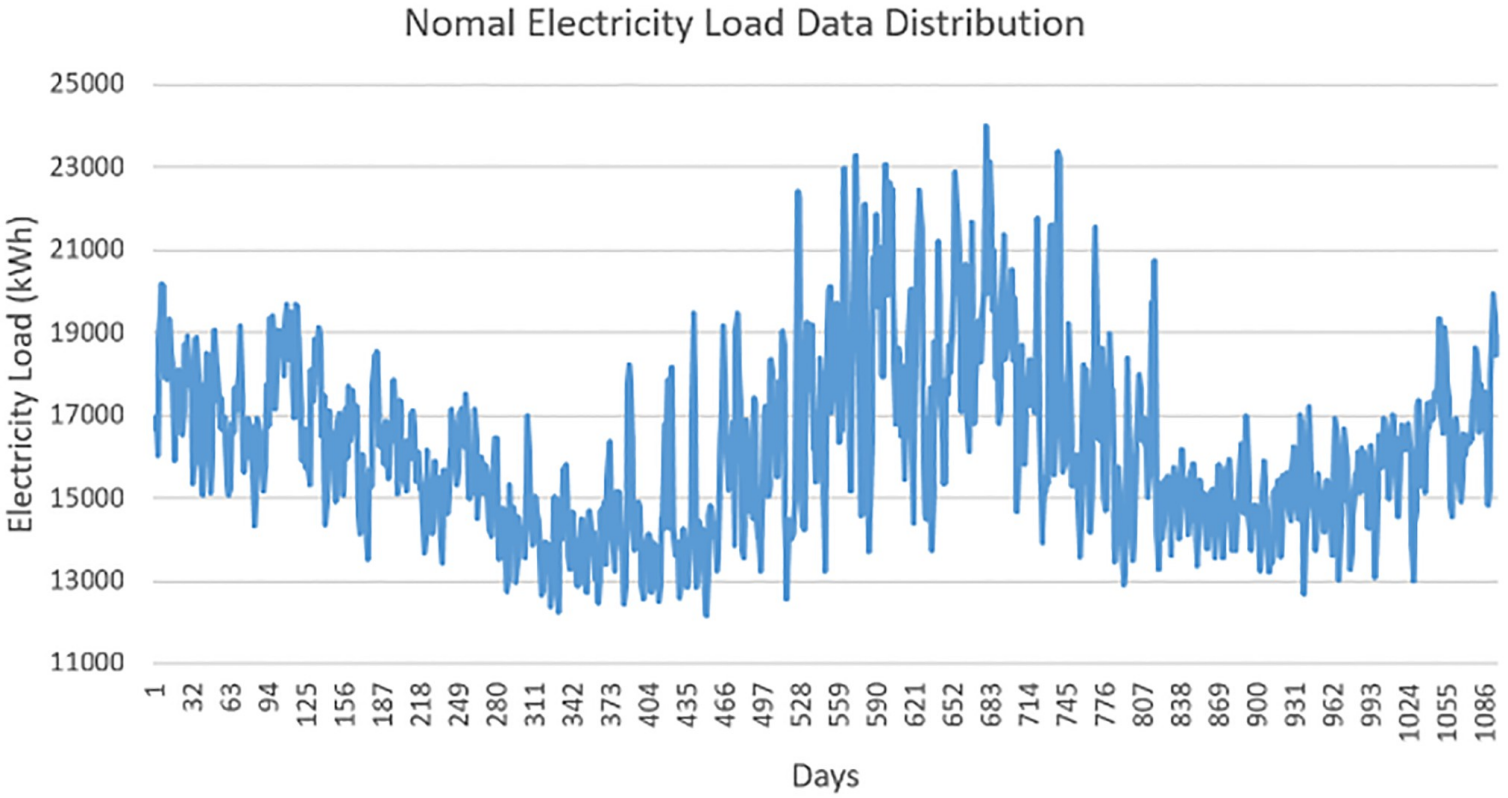
High consumers electricity load.

We have forecasted the electricity load and price for Low Consumers (LC) and High Consumers (HC). The LC consumer’s spikes pattern is low compared to the HC consumer. The upcoming/forecasted price of a market depends on the upcoming load. LC’s normal electricity load data is shown in Fig 5. We can see the LC’s electricity range is from 2000kWh to 13000kWh. The pattern of the average load is of the city of New York. Furthermore, the regular consumption pattern of HC consumers is also shown in Fig 6. The consumption of HC starts from 12000kWh and ends at nearly 24000kWh. We have forecasted the price and load for both LC and HC The modified version of ResNet-152 and SVM predicts the electricity load accurately. This will help the utility pricing market to decide the pricing according to the forecasted electricity load.

**Fig 7:**
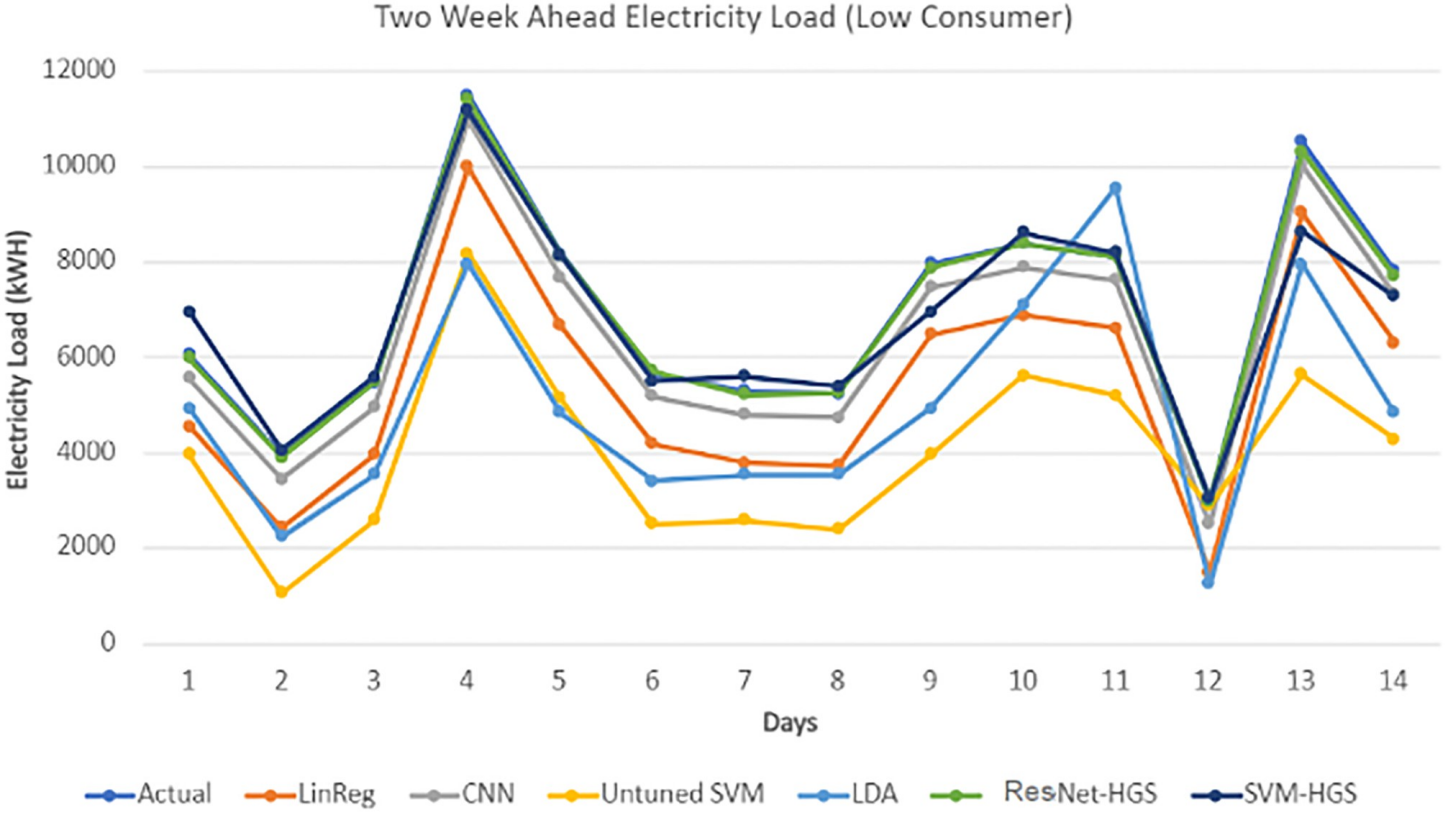
15 days LC consumer.

**Fig 8:**
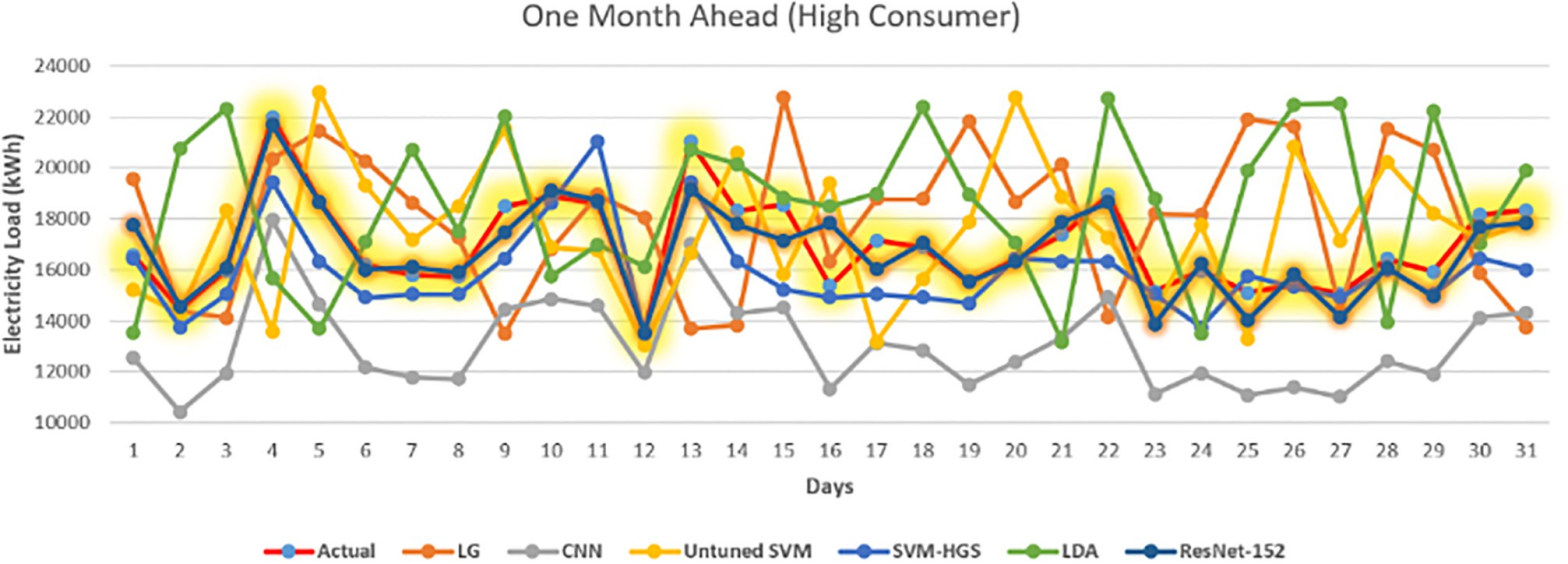
30 days HC consumer.

**Fig 9:**
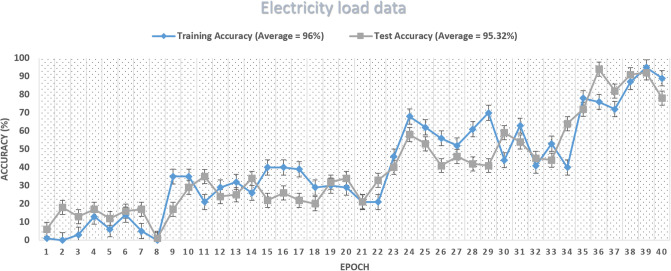
Accuracy arc of the proposed model on load data.

Furthermore, using the same methodology, we projected the upcoming power load for the next two and four weeks with high accuracy of 98%, as shown in Figs 7 and 8. We observed that our suggested method surpasses conventional approaches because of forecasting. ResNet152-HGS, the proposed algorithm, outperforms than proposed algorithm SVM-HGS. Our proposed method improved SVM outperforms the techniques in [26]. When the parameters of the ResNet method and SVM technique are tuned with HGS, our model’s training step’s accuracy gradually increases from 10 to 97%, as shown in Fig 9. At epoch 2, our model attained the training and test accuracy of 40%. While reaching the 80% training of the model, our model is trained maximum, which leads to the accuracy of 97.98% on electricity load data.

**Fig 10:**
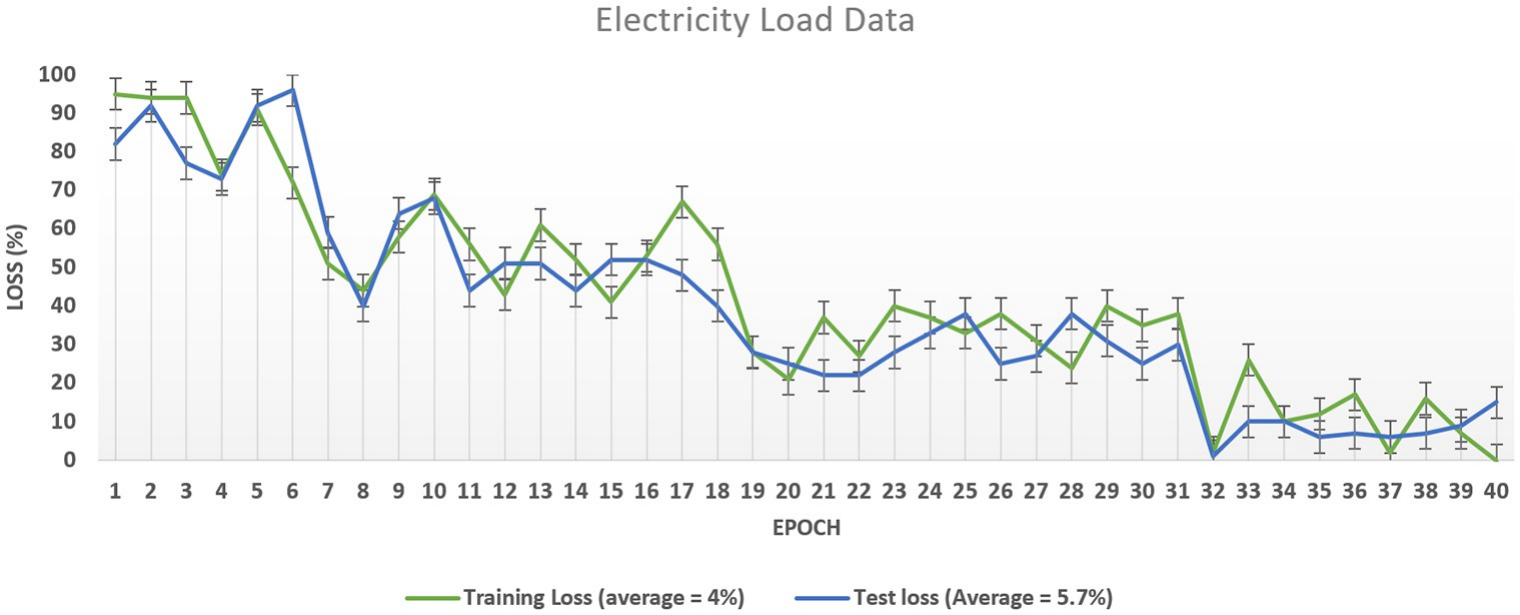
Loss arc of the proposed model on load data.

**Fig 11:**
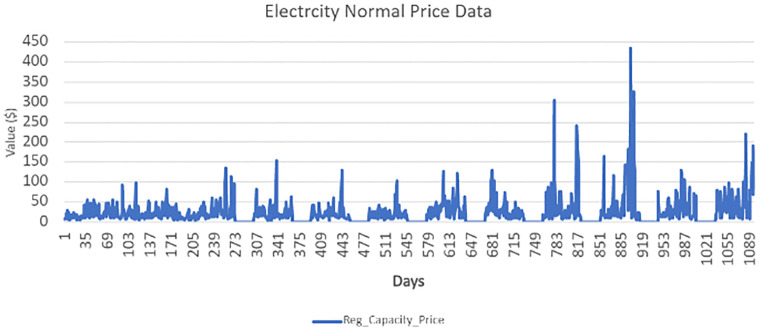
Normal pattern electricity price ISO-NE dataset 2018-2021.

**Fig 12:**
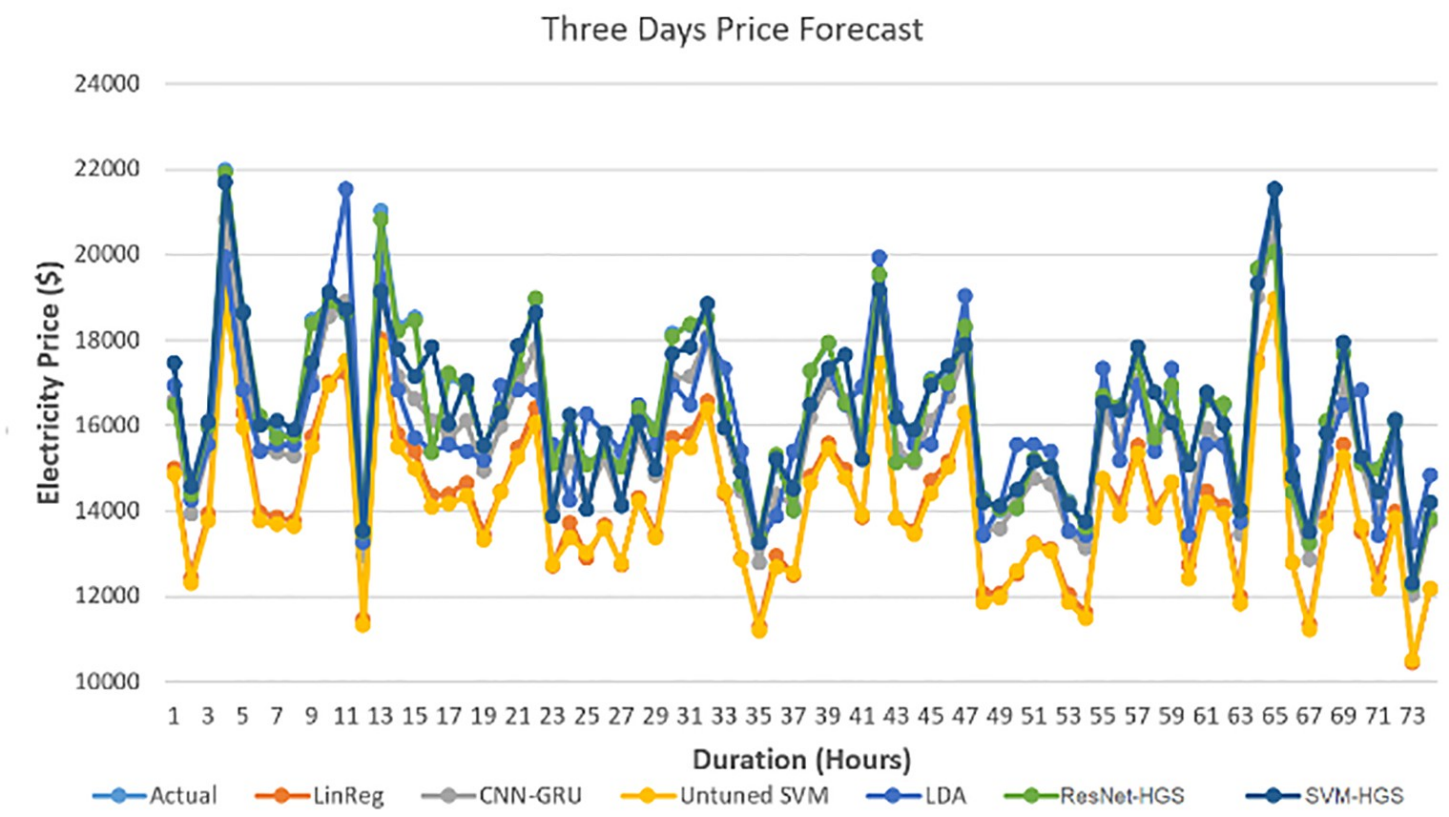
Three days electricty load forecast.

At the same time, the model loss decreases while reading to the epoch near 4. Our model’s training and testing loss become the same when its training reaches epoch three, as shown in Fig 10. It is reduced to 3% when reaching epoch 4. The high training accuracy and less model loss indicate that our optimized algorithm worked better in accurate forecasting. The hourly electricity price data duration distribution from 2018 to 2021 is shown in Fig 11. Special days have divergent price patterns. The market can get benefits with the help of upcoming electricity load and electricity prices. With the help of the same model used for the electricity LF, we have also forecasted the electricity price for 72 hours, as shown in Fig 12. The price spike at hour 12 is too low; however, at hour 4, the spikes are too high. The distribution of electricity market price is normal from hours 14 to 24. The prediction of our suggested system model is nearly accurate with respect to the actual market cost, which means our algorithm can forecast the short-term electricity price with less time complexity. We have also expanded the boundary of our testing data prediction to 72 hours. With an in-depth view of the benchmark schemes and our proposed model, the ResNet152 tuned with HGS has the better accuracy of attaining the best accuracy in the electricity PF.

Our tuned models are trained well to predict the spikes among the data, which have an advantage over the market competitors to get maximum profit in fixing the electricity price for the upcoming electricity load.

**Fig 13:**
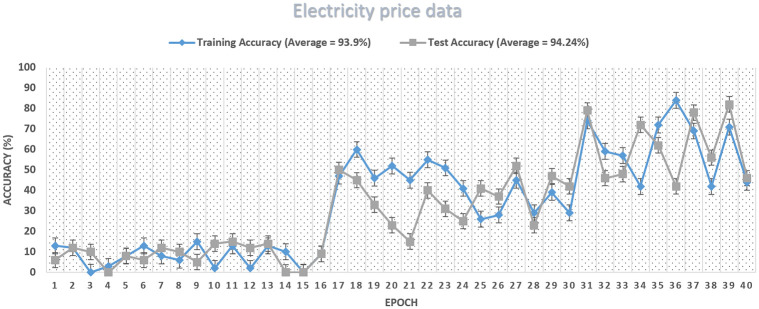
Accuracy curve of PF model.

**Fig 14:**
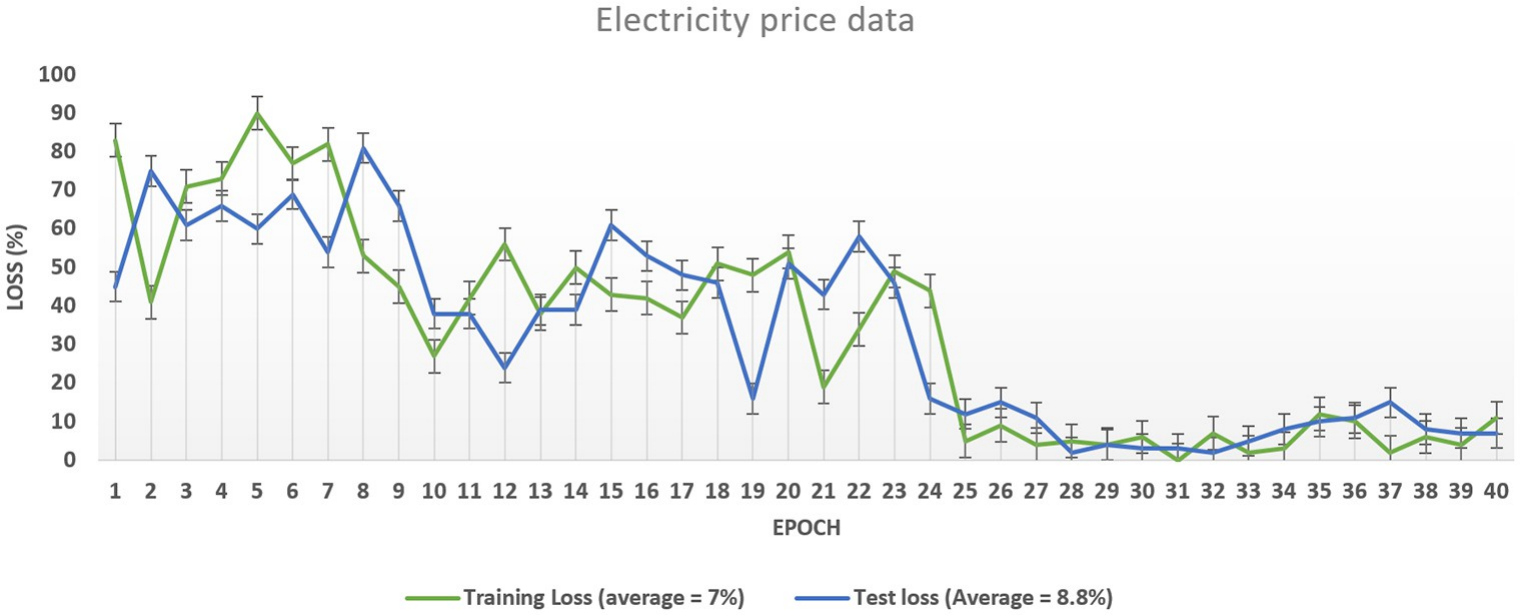
Loss curve of PF model.

As discussed above, our proposed system model’s accuracy is improved than the benchmark schemes, which means the training algorithm is achieving accuracy with the number of iterations can be seen in Fig 13. The proposed system model’s accuracy improves with the increase in epoch while the loss value decreases, as in Fig 14. Our proposed technique performs better in accuracy, attaining 93.15% and 86.26%, respectively. The accuracy increases as the model training is improved using the HGS-optimized parameter values. The model complexity is reduced at the initial stage when the features are reduced and secondly when the HGS technique provides the optimized values to the parameter of the proposed method.

**Fig 15:**
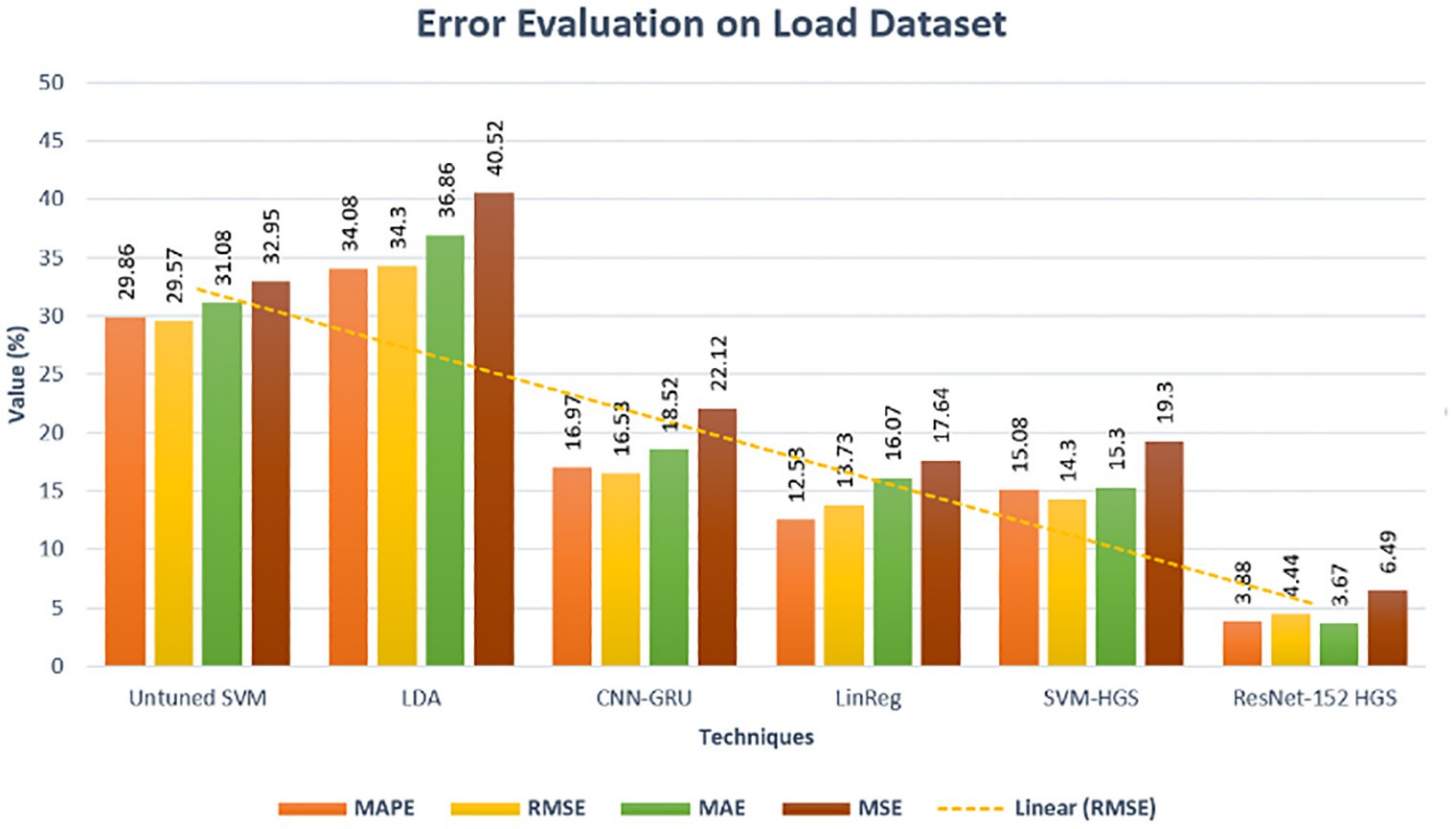
Performance error evaluation electricity load dataset.

**Fig 16:**
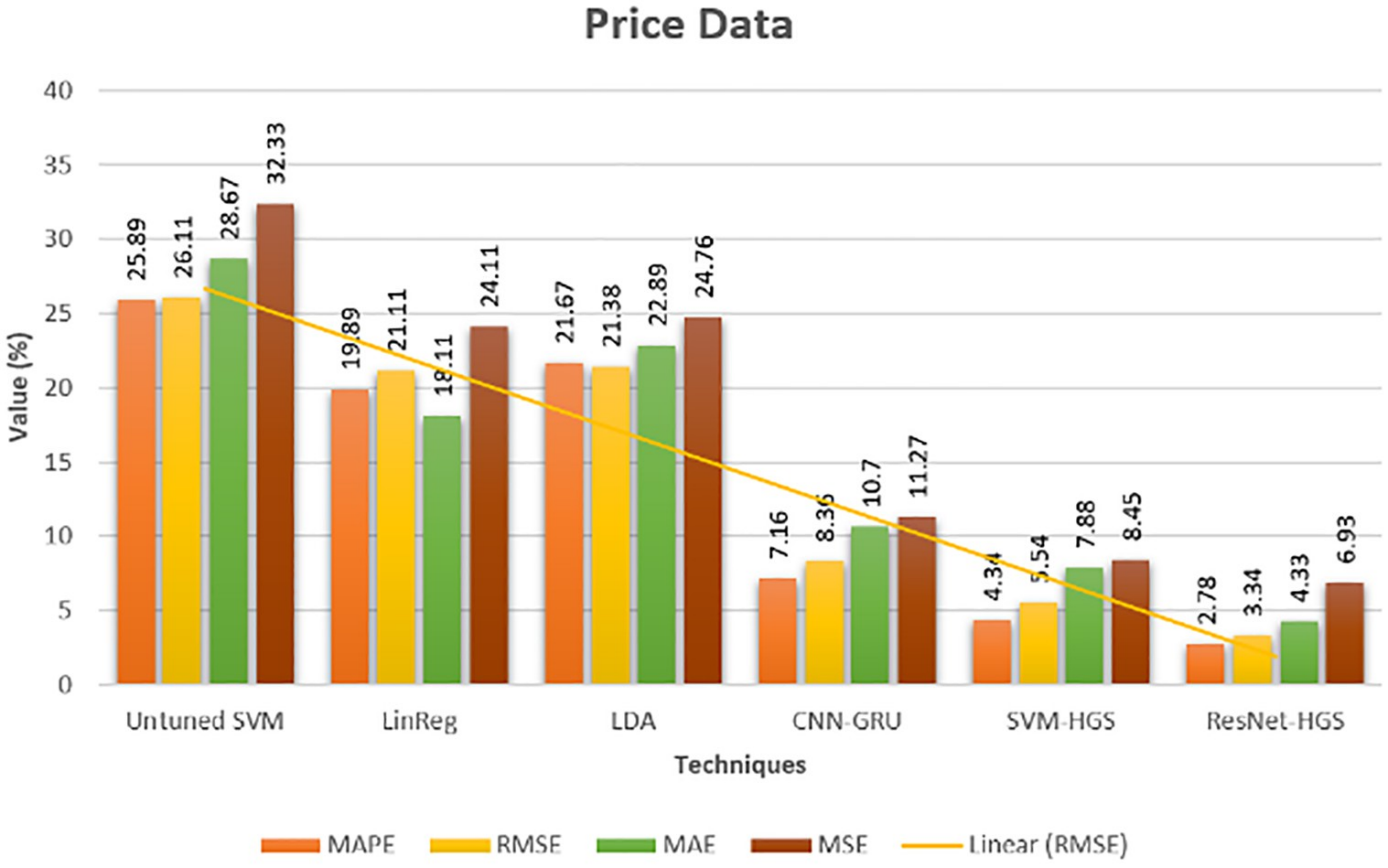
Performance error evaluation electricity price dataset.

After getting the accurate results of our proposed model, the evaluation of our model is necessary because sometimes our model shows good accuracy, but in actuality, the model overfits. We evaluated our model using Performance Error (PE) metrics to avoid such issues. The PE of ResNet-HGS on the loaded dataset is significantly less than the other existing methods, as shown in Fig 15. Fig 16 described that our model completely fits on data, which reduces the performance gap between the actual and predicted, in terms of achieving low computational cost and max precision and accuracy. The minimized error rates describe the model authenticity of better training. Our proposed model has the lowest performance error metric value than [27, 28].

**Fig 17:**
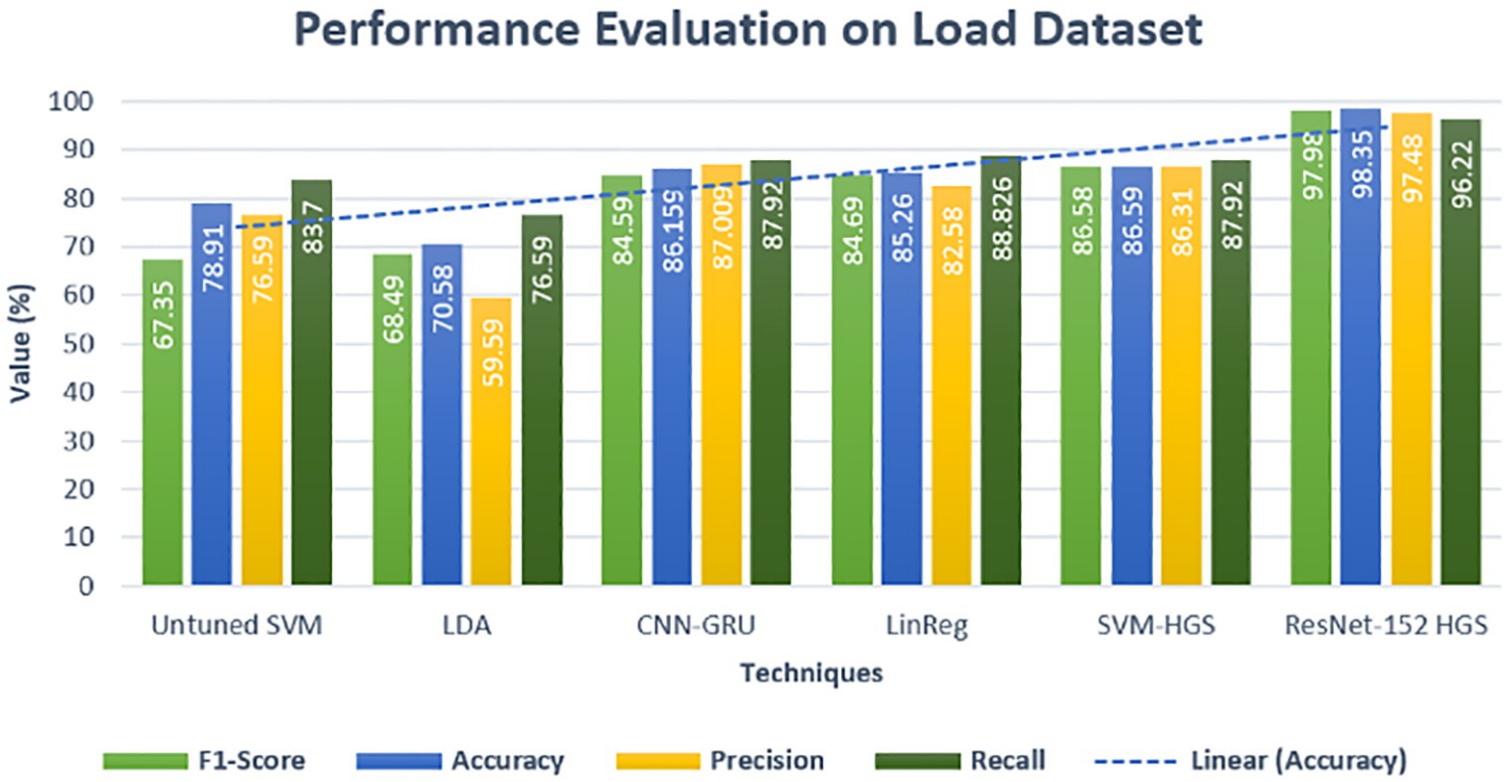
Performance evaluation electricity load dataset.

**Fig 18:**
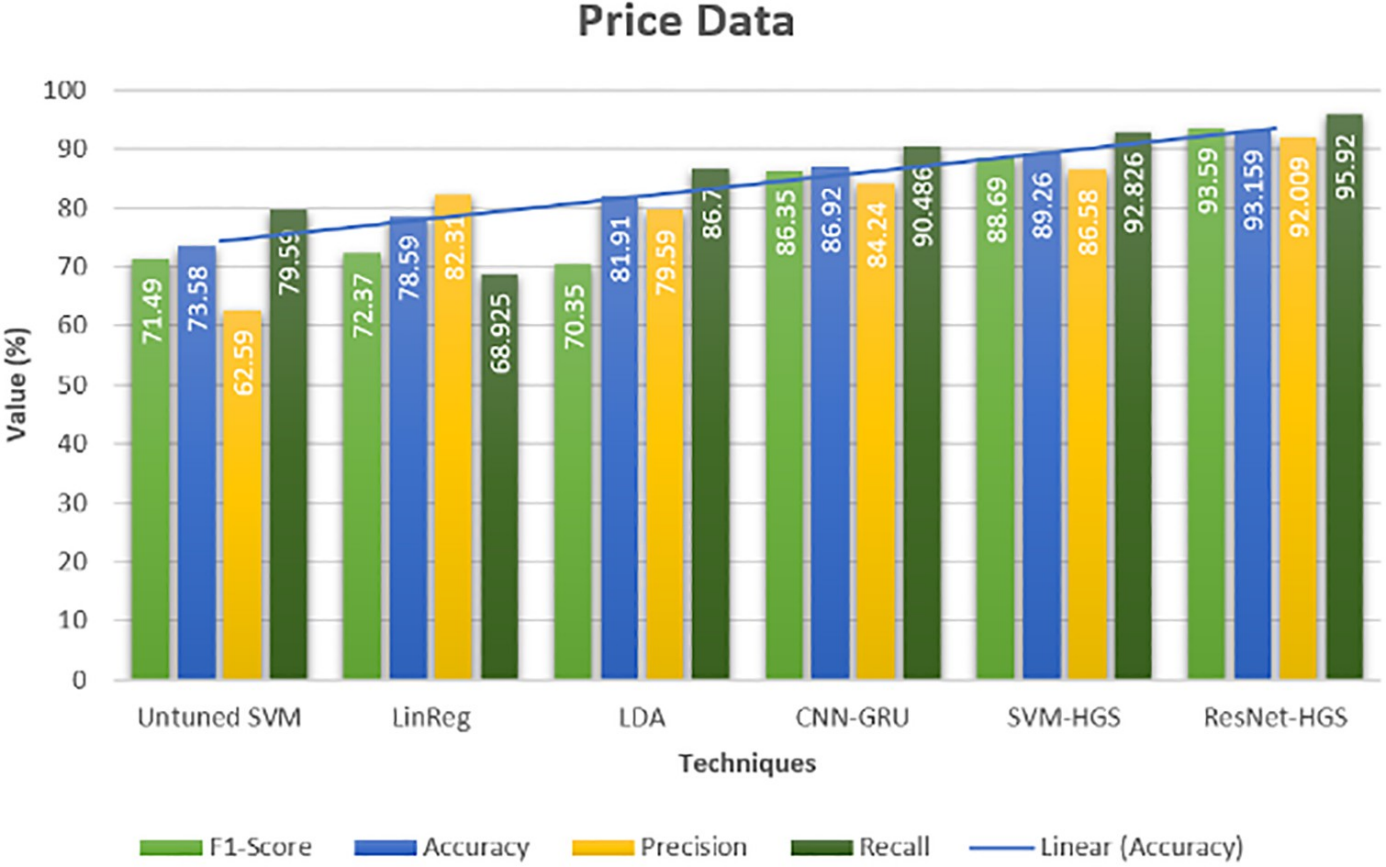
Performance evaluation electricity price dataset.

To verify the predicted model that either has reduced the chances of overfitting, we have conducted a metrics evaluation to check the error/difference between the actual and forecast data. The best separation of data, i.e., how much data is predicted correctly and falsely, precision, accuracy, F-score and recall methods are applied. The performance on the load and price datasets are shown in visual form in Figs 17 and 18. Our proposed approach also has a better classification of correct classification on price data with an accuracy of 93.159%, as shown in Figs 17 and 18. Our model’s accurate classification and good accuracy are the better parameter values of the classifiers provided by the metaheuristic approach. The accuracy of proposed techniques ResNet-152-HGS and SVM-HGS have 86.58% and 92.01% precision, which is far better than the existing approaches. Furthermore, the proposed methods also have good accuracy on the price data, which clearly shows the superiority of our proposed technique over the existing approach [28, 29]. The statistical analysis of the suggested method is demonstrated in Table 4. To assess our suggested model, we used ten statistical approaches. The superiority of the proposed model is also shown in the analysis in Table 4.

**Table 4:** Proposed approach vs benchmark algorithms (statistical analysis).

Techniques		Pearsons	Spearman’s	Kendall	Chi-Squared	Students	Paired Studentas	ANOVA	Mann-Whitney	Kruskal
ResNet152-HGS	FV	1	1	1	3932	0.2	0.98	0	37020	0
	PV	0	0	0	0	0.8	0.33	1	0.39	1
SVM-HGS	FV	1	1	1	13547	0.5	1.26	0	37447	0
	PV	0	0	0	0.18	0.6	0.21	1	0.48	1
UnTuned SVM	FV	1	1	1	107	-0.2	-0.98	0	39227	0
	P.V.	0	0	0	0.04	0.8	0.33	1	0.79	1
CNN-GRU	FV	1	1	1	73432	0	1	0	37538	0
	P.V.	0	0	0	0	1	1	1	0.5	1
LDA	FV	1	1	1	109.4	0	0.44	0	41232	0
	P.V.	0	0	0	0.05	1	0.66	1	0.81	1
LinReg	FV	1	1	1	73432	0	1	0	37538	0
	PV	0	0	0	0	1	1	1	0.5	1

where FV means F-statistic and PS means Probability value.

**Fig 19:**
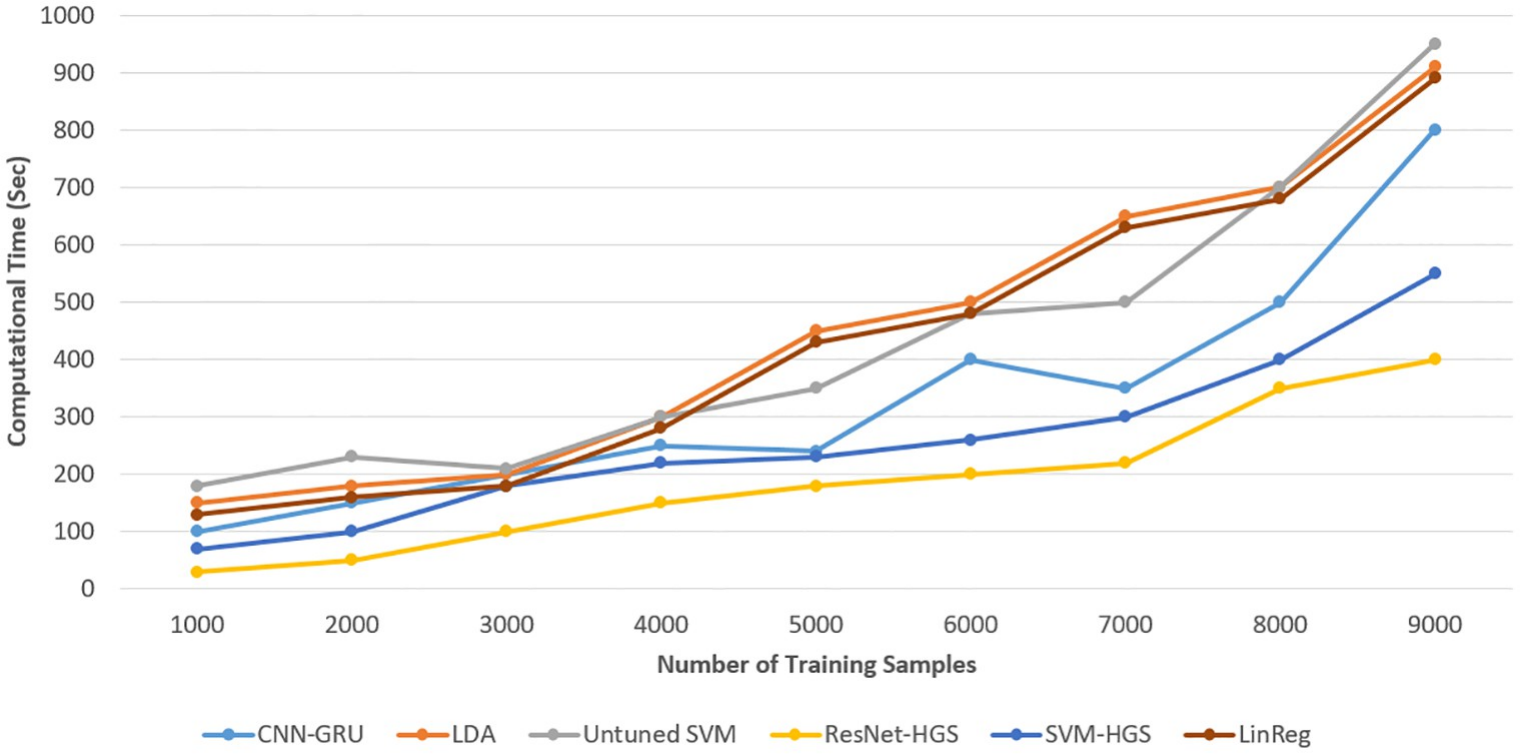
Computational complexity/time of proposed and benchmark model.

The computational complexity of the proposed system model and the benchmark is described in Fig 19. The complexity of our suggested approach is significantly lower than that of the other techniques. ResNet tuned with HGS has the shortest execution time and highest accuracy. SVM-HGS has the lowest computational complexity after ResNet152-HGS. The proposed model has an average of 15% lowest time complexity than the existing method. This demonstrates that our model forecasted the electrical load and price with the most remarkable accuracy possible than the current scheme [7, 30, 31].

## Conclusion and future work

In this work, we investigated the problems of computational complexity, processing time, non-linearity, optimum parameter values and the problem of overfitting, as well as to improve classifier performance and accuracy. The input data contains missing values, redundant features and irrelevant attributes. Therefore, feature engineering, which comprises selection and extraction, is employed to preprocess the initial input data early. The feature engineering process, which involves choosing the most relevant features, uses XGBoost, RF and RFE. The HGS optimizer provides optimal values for the classifier parameters, which improves the proposed classifiers’ performance, including ResNet and SVM. After extensive simulations, the results of our proposed model require very little computing time. Furthermore, improved preprocessing by selecting the best features, increased training accuracy, reduced model loss, high-performance accuracy and lower performance error values were achieved. A stabilization study is also performed with varied data sizes to ensure that the recommended models are stable. Our proposed model Improved ResNet152 and Improved SVM achieved 97.48% and 86.31% precision in LF while 92.01% and 86.58% precision in PF than the existing schemes as compared in the simulation section.

In the future, we plan to combine the new optimizers with an improved version of deep learning and machine learning approaches to handle large amounts of data quickly with maximum accuracy. We will use improved methods to analyze commercial and industrial data. We will also consider medium- and long-term load and price forecasts.

## References

[1] Butt OM, Zulqarnain M, Butt TM. Recent advancement in smart grid technology: Future prospects in the electrical power network. Ain Shams Engineering Journal. 2021;12(1):687–695. doi: 10.1016/j.asej.2020.05.004

[2] Haben S, Singleton C, Grindrod P. Analysis and clustering of residential customers’ energy behavioral demand using smart meter data. IEEE Transactions on Smart Grid. 2015;7(1):136–144. doi: 10.1109/TSG.2015.2409786

[3] Padhi DK, Padhy N, Bhoi AK, Shafi J, Ijaz MF. A fusion framework for forecasting financial market direction using enhanced ensemble models and technical indicators. Mathematics. 2021;9(21):2646. doi: 10.3390/math9212646

[4] Dai Y, Zhao P. A hybrid load forecasting model based on support vector machine with intelligent methods for feature selection and parameter optimization. Applied Energy. 2020;279:115332. doi: 10.1016/j.apenergy.2020.115332

[5] Dong Y, Ma X, Fu T. Electrical load forecasting: A deep learning approach based on K-nearest neighbors. Applied Soft Computing. 2021;99:106900. doi: 10.1016/j.asoc.2020.106900

[6] Fekri MN, Patel H, Grolinger K, Sharma V. Deep learning for load forecasting with smart meter data: Online Adaptive Recurrent Neural Network. Applied Energy. 2021;282:116177. doi: 10.1016/j.apenergy.2020.116177

[7] Jalali SMJ, Ahmadian S, Khosravi A, Shafie-khah M, Nahavandi S, Catalão JPS. A novel evolutionary-based deep convolutional neural network model for intelligent load forecasting. IEEE Transactions on Industrial Informatics. 2021;17(12):8243–8253. doi: 10.1109/TII.2021.3065718

[8] Vivian J, Prataviera E, Cunsolo F, Pau M. Demand Side Management of a pool of air source heat pumps for space heating and domestic hot water production in a residential district. Energy Conversion and Management. 2020;225:113457. doi: 10.1016/j.enconman.2020.113457

[9] Han Z, Sun X, Wei H, Ji Q, Xue D. Energy saving analysis of evaporative cooling composite air conditioning system for data centers. Applied Thermal Engineering. 2021;186:116506. doi: 10.1016/j.applthermaleng.2020.116506

[10] Ahmad W, Ayub N, Ali T, Irfan M, Awais M, Shiraz M, et al. Towards short term electricity load forecasting using improved support vector machine and extreme learning machine. Energies. 2020;13(11):2907. doi: 10.3390/en13112907

[11] Wang X, Palazoglu A, El-Farra NH. Operational optimization and demand response of hybrid renewable energy systems. Applied Energy. 2015;143:324–335. doi: 10.1016/j.apenergy.2015.01.004

[12] Elkazaz M, Sumner M, Thomas D. Energy management system for hybrid PV-wind-battery microgrid using convex programming, model predictive and rolling horizon predictive control with experimental validation. International Journal of Electrical Power & Energy Systems. 2020;115:105483. doi: 10.1016/j.ijepes.2019.105483

[13] McPherson M, Stoll B. Demand response for variable renewable energy integration: A proposed approach and its impacts. Energy. 2020;197:117205. doi: 10.1016/j.energy.2020.117205

[14] Pilo F, Pisano G, Ruggeri S, Troncia M. Data analytics for profiling low-voltage customers with smart meter readings. Applied Sciences. 2021;11(2):500. doi: 10.3390/app11020500

[15] Logenthiran T, Srinivasan D, Shun TZ. Demand side management in smart grid using heuristic optimization. IEEE Transactions on Smart Grid. 2012;3(3):1244–1252. doi: 10.1109/TSG.2012.2195686

[16] Khashei M, Chahkoutahi F. Electricity demand forecasting using fuzzy hybrid intelligence-based seasonal models. Journal of Modelling in Management. 2022;17(1):154–176. doi: 10.1108/JM2-06-2020-0159

[17] Yousaf A, Asif RM, Shakir M, Rehman AU, S. Adrees M. An improved residential electricity load forecasting using a machine-learning-based feature selection approach and a proposed integration strategy. Sustainability. 2021;13(11):6199. doi: 10.3390/su13116199

[18] Azeem A, Ismail I, Jameel SM, Harindran VR. Electrical load forecasting models for different generation modalities: a review. IEEE Access. 2021;9:142239–142263. doi: 10.1109/ACCESS.2021.3120731

[19] Zhang Z, Hong WC. Electric load forecasting by complete ensemble empirical mode decomposition adaptive noise and support vector regression with quantum-based dragonfly algorithm. Nonlinear dynamics. 2019;98:1107–1136. doi: 10.1007/s11071-019-05252-7

[20] Liao Z, Pan H, Huang X, Mo R, Fan X, Chen H, et al. Short-term load forecasting with dense average network. Expert Systems with Applications. 2021;186:115748. doi: 10.1016/j.eswa.2021.115748

[21] Atef S, Ismail N, Eltawil AB. A new fuzzy logic based approach for optimal household appliance scheduling based on electricity price and load consumption prediction. Advances in Building Energy Research. 2022;16(2):262–280. doi: 10.1080/17512549.2021.1873183

[22] Chen Z, Chen Y, Xiao T, Wang H, Hou P. A novel short-term load forecasting framework based on time-series clustering and early classification algorithm. Energy and Buildings. 2021;251:111375. doi: 10.1016/j.enbuild.2021.111375

[23] Wen L, Li X, Gao L. A transfer convolutional neural network for fault diagnosis based on ResNet-50. Neural Computing and Applications. 2020;32:6111–6124. doi: 10.1007/s00521-019-04097-w

[24] Zainab A, Syed D, Ghrayeb A, Abu-Rub H, Refaat SS, Houchati M, et al. A multiprocessing-based sensitivity analysis of machine learning algorithms for load forecasting of electric power distribution system. IEEE Access. 2021;9:31684–31694. doi: 10.1109/ACCESS.2021.3059730

[25] Pal S, Singh N, Chaudhary NK. Correlation-Based Short-Term Electric Demand Forecasting Using ANFIS Model. In: Machine Learning, Advances in Computing, Renewable Energy and Communication: Proceedings of MARC 2020. Springer; 2022. p. 471–482.

[26] Hafeez G, Alimgeer KS, Khan I. Electric load forecasting based on deep learning and optimized by heuristic algorithm in smart grid. Applied Energy. 2020;269:114915. doi: 10.1016/j.apenergy.2020.114915

[27] Salkuti SR. Short-term electrical load forecasting using radial basis function neural networks considering weather factors. Electrical Engineering. 2018;100(3):1985–1995. doi: 10.1007/s00202-018-0678-8

[28] Zhou Y, Wang J, Li Z, Lu H. Short-term photovoltaic power forecasting based on signal decomposition and machine learning optimization. Energy Conversion and Management. 2022;267:115944. doi: 10.1016/j.enconman.2022.115944

[29] Xu A, Tian MW, Firouzi B, Alattas KA, Mohammadzadeh A, Ghaderpour E. A New Deep Learning Restricted Boltzmann Machine for Energy Consumption Forecasting. Sustainability. 2022;14(16):10081. doi: 10.3390/su141610081

[30] Aslam S, Ayub N, Farooq U, Alvi MJ, Albogamy FR, Rukh G, et al. Towards electric price and load forecasting using cnn-based ensembler in smart grid. Sustainability. 2021;13(22):12653. doi: 10.3390/su132212653

[31] Rafi SH, Deeba SR, Hossain E, et al. A short-term load forecasting method using integrated CNN and LSTM network. IEEE Access. 2021;9:32436–32448. doi: 10.1109/ACCESS.2021.3060654

