# Expression of Concern: Ensemble learning approach for advanced metering infrastructure in future smart grids

**DOI:** 10.1371/journal.pone.0345142

**Published:** 2026-03-18

**Authors:** The PLOS One Editors

The *PLOS One* Editors issue this Expression of Concern for this article [1] because of concerns identified about the peer review process. Readers are advised to interpret the article [1] with caution.
